# An electrical load measurements dataset of United Kingdom households from a two-year longitudinal study

**DOI:** 10.1038/sdata.2016.122

**Published:** 2017-01-05

**Authors:** David Murray, Lina Stankovic, Vladimir Stankovic

**Affiliations:** 1 Department of Electronic and Electrical Engineering, University of Strathclyde, Glasgow G1 1XW, UK

**Keywords:** Electrical and electronic engineering, Energy and behaviour, Sustainability, Energy modelling

## Abstract

Smart meter roll-outs provide easy access to granular meter measurements, enabling advanced energy services, ranging from demand response measures, tailored energy feedback and smart home/building automation. To design such services, train and validate models, access to data that resembles what is expected of smart meters, collected in a real-world setting, is necessary. The REFIT electrical load measurements dataset described in this paper includes whole house aggregate loads and nine individual appliance measurements at 8-second intervals per house, collected continuously over a period of two years from 20 houses. During monitoring, the occupants were conducting their usual routines. At the time of publishing, the dataset has the largest number of houses monitored in the United Kingdom at less than 1-minute intervals over a period greater than one year. The dataset comprises 1,194,958,790 readings, that represent over 250,000 monitored appliance uses. The data is accessible in an easy-to-use comma-separated format, is time-stamped and cleaned to remove invalid measurements, correctly label appliance data and fill in small gaps of missing data.

## Background & Summary

Smart meter roll-outs have been implemented or planned across the world to better manage residential energy demand, conserve energy, improve billing accuracy, and help users understand the energy implications of their appliance usage habits. For example, by 2020, it is expected that almost 72% of European consumers will have a smart meter to comply with EU energy market legislation in the Third Energy Package, requiring all EU member states, that have obtained positive results from the economic analysis, to implement smart metering^1^. In most countries, regulations require that every smart meter is bundled with an in-home display (IHD), providing real-time consumption information^2–4^. The IHD will access live and historical energy data, providing feedback beyond the current capabilities of suppliers. The IHDs and communications connectivity of the new meters enable new types of services, such as:

- energy feedback via non-intrusive appliance load monitoring, i.e., the ability to extract consumption of individual appliances from the household aggregate^5–7^;
- appliance usage monitoring, i.e., analysing the performance of an appliance under real-life usage conditions to inform appliance life-cycle analysis or provide feedback on energy efficient usage^8^;
- load shifting, i.e., exploiting flexibility in time-of-use of appliances to manage peak demand, with the incentive of lower tariffs and improve demand response^9^;
- retrofit advice, i.e., installing replacement appliances or energy savings measures^10^;
- smart home automation, for improving energy conservation, comfort and security in the home^11^;
- activity recognition, i.e., analysing energy consumption through the lens of activities, potentially more meaningful to users^12^.

In order to develop and test analytical methods to support these services, smart meter-style data are necessary, generated in a real-world setting, instead of laboratory conditions, where householders uninterruptedly perform their normal routines.

Currently, there are a number of open source datasets available (see Table 1) which provide such data with varying levels of detail and scale.

The REFIT (*Personalised Retrofit Decision Support Tools For UK Homes Using Smart Home Technology*) Project Electrical Load Measurements Dataset, presented in this paper, stands apart from other available datasets, as it closely mimics the data that will be available via the SMETS2 smart meter standard^2^ which will provide active power data at a sample rate of 10 s. Other datasets, such as REDD^13^ and BLUED^14^, have sampled data in excess of 10 KHz while only recording for a few weeks; others, such as HES^15^, AMPds^16,17^ and IHEPCDS^18^ have recorded for at least a year, but at sampling intervals of 1 min or more. The UK-Dale dataset^19^ sampled the aggregate current and voltage of 3 homes at 16 KHz and 2 homes at 1 Hz, but individual appliances at 6 s—the five homes were monitored for different periods from 39 to 786 days. Other publicly available datasets focus on capturing many individual appliance signatures, e.g., ACS-F2 (ref. 20) and Tracebase^21^, while many datasets offer aggregate and submetering measurements, sampled at 1 Hz, such as the ECO^22^ and Smart* (ref. 23), from fewer than 10 houses.

The REFIT Electrical Load Measurements dataset, on the other hand, contains data from 20 homes, for a continuous period of around two years; this makes this dataset the only such UK-based dataset at sampling rate greater than 1 min that combines large scale (20 homes all monitored at the same sampling rate) and long duration. The dataset comprises the active power measurements of the household aggregate as well as 9 appliances, all recorded at 8-second intervals. During the monitoring period, the households were conducting their usual domestic activities.

The data monitoring platform and database design were presented in the conference paper ‘A data management platform for personalised real-time energy feedback’^24^. This paper significantly builds upon the conference version by providing a detailed description of the whole dataset, including analysis about the quality of the data recorded, how the dataset was cleaned and labelled, and how the cleaned data can be used. This data is invaluable to scientists broadly involved in energy analysis, low carbon agenda, energy efficiency feedback and policy. The data has already been used to support algorithmic work pertaining to load disaggregation^6^, temporal dynamics of demand response^9^, appliance modelling and usage patterns^8^, and linking domestic routines to their energy implications^12^.

## Methods

### Selection methodology

The homes participating in the REFIT study were recruited via email and leaflet drops. In total, 57 households replied with basic information about their household. Final selection was based on the householder’s familiarity with information and communication technology (ICT) as well as a mix of household occupancy, including retired couples, working couples and families with children ranging from infants to young adults. Some houses were excluded for a number of reasons, mainly related to connectivity, such as utility meters being underground meaning that signal acquisition would be difficult, or absence of a broadband connection^10^. Occupancy and physical characteristics of each house relevant to electricity consumption is shown in Table 2. Ethics approval was granted by Ethics Approvals (Human Participants), Sub-committee, Research Office, Loughborough University and all participants gave informed consent and understood how their data would be used.

In each house, nine appliances were selected to be monitored via plug meters. Appliance selection was motivated by the completed Household Electricity Survey (HES)^15^, a large study conducted by the UK’s Department of Environment and Climate Change (DECC). Since the HES study focused on collecting a large amount of data about consumer attitudes towards energy consuming practices and energy demand, the study prioritised appliances with relatively high electrical consumption and/or frequent use to be monitored.

With respect to the monitoring priorities from HES (see Appendix II of the HES report^15^), the main appliances from the energy demand point of view are cold appliances (refrigerators, freezers, fridge-freezers, etc.), cooking appliances (microwaves, kettles, etc.), ICT (computers, screens, printers, consoles, etc.), utility room appliances (washing machines, tumble dryers, dishwashers), while low priority items include mobile phone chargers, hair straighteners and similar small items which may not be used regularly or moved frequently.

In the REFIT study, this HES prioritization list was used as a guide when selecting appliances to monitor, unless study participants explicitly requested monitoring unusual appliances, such as a vivarium or pond pump. Table 3 lists all appliances monitored in each house. Column 4 in Table 2 shows the total number of electrical appliances in each house according to the house survey obtained at the beginning of the study. Note that all REFIT study houses used gas central heating systems as primary source of fuel and there were no other HVAC systems present.

### Monitoring set-up

**Figure 1:**
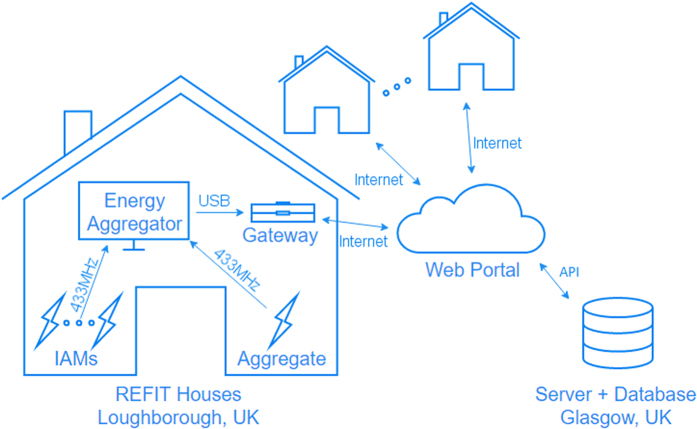
REFIT remote real-time data collection schematic.

Figure 1 shows the schematic of the data collection platform. To ensure reliability, scalability and performance, all equipment used in the REFIT study was commercial off-the-shelf hardware available for purchase at the beginning of the study.

Energy sensors (ten in each house) wirelessly sent readings every 8 s to an energy aggregator, which was connected to a communications gateway. The gateway, connected to the broadband router, forwarded readings to the web portal in the cloud. From the web portal, the data was requested by our server in Glasgow and stored in a MySQL database.

The overall platform is designed to be as similar as possible to a typical smart meter installation^2^ in terms of data collection and in-home presence. Indeed, the used aggregator comes with an IHD that displays usage information and basic historical statistics similar to what will be available after smart meter rollout^2^. However, we note that individual appliance monitors (IAMs) will not be part of the smart meter installation^2^, but they are helpful to correlate use times and power usage, design, model, test and validate analytical approaches.

In the following, we describe each component of the monitoring platform.

### Household aggregate

The most important measurement in each house was the household aggregate energy consumption as this will imitate what smart meters will be able to provide. The household aggregate was measured by a CurrentCost transmitter (specifications available at http://www.currentcost.com/product-transmitter.html), which contains a single phase current clamp and a transmission module which wirelessly transmits readings every 8 s using Radio Frequency (RF) 433 MHz to the energy aggregator. The aggregator used was a CurrentCost EnviR module that also contains an IHD.

CurrentCost monitoring equipment has been used successfully in many previous trials, e.g., in trials^19,25–30^, which is why it was chosen over other options available on the market around the time the study started. It should be noted that the sensor does not measure mains voltage, thus there may be variation in the Watts value generated. The manufacturer did not give any details with regards to the internal workings of their sensors, however testing quantifies their relative error of around 6% (ref. 19).

Six homes in the study had solar panels installed. In three cases rewiring was done to remove the effect of solar panel generation (Houses 1, 6 & 7). In the other three (Houses 3, 11 & 21) rewiring was not possible and the aggregate of these houses was recorded as is with solar interference. As the sensor used to measure aggregate energy consumption was unable to distinguish the direction of power flow, solar panels appeared as additional power consumption resulting in a bell-curve-shaped power consumption increase during the day with significant noise due to weather changes, such as clouds.

### Individual appliance monitors (IAM)

Each house was supplied with 9 CurrentCost IAMs, which is the maximum number supported by the associated EnviR module without the likelihood of causing data loss from transmission collisions. Each IAM provided the power consumption (in Watts) for each appliance which was connected, at a sampling rate of 8 s. All IAM readings were collected via the EnviR aggregator which was then connected to the communications gateway.

Similarly to the household aggregate, the IAMs only monitor the current and not the voltage which means there may be a variation in the supply voltage which introduces an error in the reading. By default, all of the installed load monitoring devices had the voltage pre-set to 240 V, suitable for the UK where mains voltage is rated at 230 V +10 to −6%.

**Figure 2:**
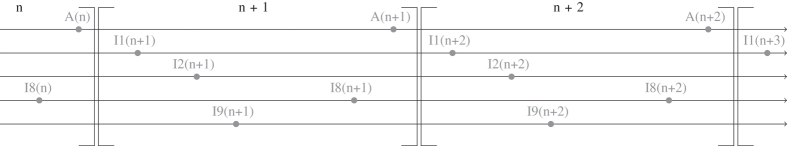
Each circle represents a reading taken. Each line represents a sensor, A being the aggregate and I1 representing IAM1 and so on. The numbers in the bracket represent sample number.

IAMs were only capable of broadcasting their readings, which results in the readings not being synchronised with the aggregate readings (discussed in the GitHub page https://github.com/JackKelly/rfm_edf_ecomanager/wiki, the type of plug used was the ‘CC_TX’). The timestamp assigned to data was the UNIX timestamp when the data was received at the Strathclyde server. Since a data request grabs data from the aggregator, which is the aggregate and last broadcasts from all the IAMs, the timestamp is the sampling time of the aggregate reading. Thus, all of the IAMs received at timestamp n+1 will be lagging the aggregate reading by up to the time since the last sample (n), that is, the offset between IAM I and Aggregate A readings will always satisfy n<offset<n+1, where n and n+1 are two consecutive sampling time of aggregate reading. See Subsections ‘Code Availability’ and ‘Known Issues’. Figure 2 shows a time representation of the readings from the Current Cost system. Note that each sampling time period is of 8 s length.

The appliances monitored were recorded during initial installation and households were advised not to unplug or move monitors during the monitoring period without notifying the REFIT project team. Any changes in the appliance monitored by an IAM that occurred during the trial are recorded in detail in the ReadMe file included with the dataset. An example of this would be House 10, which moved IAM 2 from a Freezer to a Toaster on 25/06/2014.

### Energy aggregator

The EnviR aggregator with an IHD came bundled with the CurrentCost transmitter used for measuring the household aggregate. The EnviR (http://www.currentcost.com/product-envir.html) ties all of the CurrentCost devices together acting an an energy aggregator. Its display provides information about all of the CurrentCost devices which were installed with a simple interface using buttons as navigation. Together with the transmitter for aggregate measurements this pairing best represented the combination of smart meter and IHD that would be supplied as part of the UK roll out. The EnviR communicated via a USB cable to the communications gateway allowing data to be recorded remotely.

### Communications gateway

The communications gateway used in the REFIT project for electrical measurements was the Vera3 smart home controller (http://getvera.com/controllers/vera3/). All sensors reported data wirelessly to the EnviR which then forwarded information to Vera3 using a USB connection. Finally, Vera3 sent the data to the cloud, which was an on-line dashboard available via the Vera Control (formally MiCasaVerde) on-line portal (https://cp.mios.com/login.php). Vera3 has a number of interfaces to enable additional monitoring with the following technologies: WiFi, USB, LAN, and Z-Wave. In the REFIT study, Vera3 was connected to a home broadband router via its LAN interface, to EnviR via the USB interface, and to additional sensors (measuring temperature, humidity, light intensity) via the Z-Wave interface.

### Web portal & data collection

Data collected through the communications gateway was available on the web portal via an application programming interface (API). The API requests are available remotely and all REFIT houses were linked to a single web portal account with a user account for each household (so that household could benefit from basic energy feedback available via the web portal) as well as an administration account. The list of available requests can be found at http://wiki.micasaverde.com/index.php/Luup_Requests, http://wiki.micasaverde.com/index.php/UI_Notes. Responses are given by default in the JavaScript Object Notation (JSON) formatting language.

The simplest set-up was to make a call for each sensor in a house individually. However, there are several issues with this configuration: (1) the number of requests being sent from the same account ID will be over 200, i.e., every 8 s (20 houses×10 sensors per house), (2) if the CurrentCost is reset or connections to IAMs lost, different ID numbers could be assigned to these IAMs. To eliminate this possibility our python scripts requested only sensor values which had changed and used the sensor’s universally unique identifier. This method was more robust as only 20 API calls were made every 8 s. Furthermore, this reduced bandwidth and storage requirements.

### Server & database

Requests for new data were issued to the web portal and the replies were recorded on the MySQL server hosted at the University of Strathclyde, Glasgow UK, with the following specification:

DELL PowerEdge R320 with an Intel Xeon E5-2407 and 16 gigabytes of RAM, running on Linux Debian V8.3 with MySQL 5.5.47.

Checking the connectivity of houses was done via a web page which displayed time passed from the last insert for each house by the Readings.py script. Any home which had not updated recently would show a large time difference and that home was then be contacted to check if the any of the in-home kit (aggregate sensor, IAMs, aggregator, gateway) had been inadvertently moved or unplugged. A similar page was constructed which showed all IAMs.

Initially code had been written in the Perl programming language, which was subsequently replaced with Python code due to Python’s popularity and versatility.

### Code availability

The code used to collect and check data and monitor the collection process is available at https:/github.com/David-Murray/REFIT. The code runs using Python 3 on a Debian server. The time-based job scheduler CRON, available on most Unix distributions, is used to run some segments of code at particular time intervals. The following python scripts are available:

HouseUpdater.py: This python script was responsible for keeping the information about the houses monitored up to date including the server address which API should be made to; this was run hourly via CRON.

SensorUpdater.py: This python script kept the list of sensors within the houses up to date; this would also record when the sensor was last checked which helped to show any sensors that were no longer available.

ReadingsTaskMaster.py: A python script which generated child processes for each house (see Readings.py); once each house’s script was running it would check for new houses that had been added/changed or had come back on-line and would restart their Readings.py script if required.

Readings.py: A python script that would run continuously querying and inserting sensor values into the database every 8 s. The reading time was determined by the time at which the server received the response from the API call. As IAMs were only able to broadcast their readings every 8 s they will not be synchronised with the reading of the aggregate. This means that the time associated with a record may have IAM readings up to 7 s old.

### Known issues

- CurrentCost IAMs: Occasionally, IAMs reported readings much greater than the maximum load for standard household appliances, i.e., 4,000 Watts (W), due to sensor malfunctioning. These readings were removed from the raw data.
- CurrentCost IAMs: Reporting time synchronization—although data was recorded at set intervals for all devices, the time between when IAMs reported a reading will not be in synchronization to the current second and therefore may show a mismatch to the aggregate.
- Houses’ 3, 11 & 21 aggregate readings are affected by solar panel generation as re-wiring was not possible.
- In some cases the step change in values will differ between IAM and Aggregate, as the CurrentCost system did not monitor other variables to adjust for voltage or phase angle this is caused by inductive and capacitive loads.

## Data Records

The REFIT Electrical Load Measurements’ Dataset is available in the form of CSV files. Each house has one associated CSV file containing all aggregate and IAM measurements for the entire monitoring period.

The data has been cleaned by correcting the date/time due to British Summer Time (BST), removing IAM spikes of greater than 4,000 W, and forward filling gaps of less than 2 min with previous values or, if the gap is larger than 2 min, filling with zeros, and moving data streams where appliances had been switched between plugs so there is a continuous record of each appliance (see Algorithm 1).

**Data**: Time, Power

**Result**: Forward fill NaN values of time gaps less than <2 min

Start;

**for** *n*←2 **to** *length* (*Power*) **do**

**if** *time*(*n*)*-time*(*n-1*)*<120 s* **then**

*power (n)=power* (*n−*1);

**else**

*power* (*n*)*=*0

**end**

**end**

**Algorithm 1**: Pseudocode for removing Not-a-Number (NaN) values in data.

Datetime is in the format Year (YYYY)-Month(MM)-Day(DD)

hours(HH):minutes(mm):seconds(SS). The CSV files have the following columns:

- DateTime [YYYY-MM-DD HH:mm:SS]
- UNIX Timestamp
- Aggregate [W]
- IAMs 1–9 [W].

A ReadMe TXT file is also included to provide additional information about the dataset. This includes a list of the appliances (including make & model where known) attached to each IAM as they were set-up by the REFIT team and subsequent changes that were discovered via visits to households, by being informed that an appliance had been removed/replaced or by visual inspection followed by querying the household. The format of the ReadMe file is the following:

- Introduction to the dataset
- Licensing information
- Naming scheme
- File formats
- Appliance list per house including changes made during the monitoring period and make and model where known.

**Figure 3:**
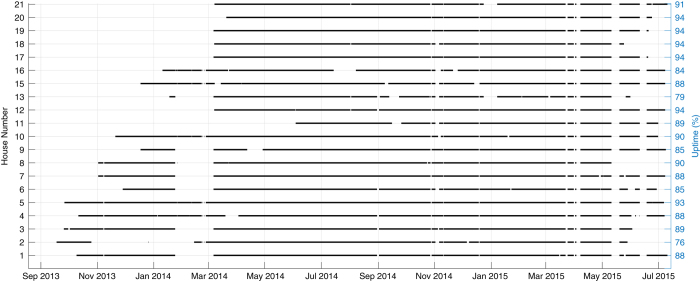
REFIT Data Availability. Gaps in the line represents an area where data was unavailable for more than a quarter of a day.

The data availability for all REFIT houses can be seen in Fig. 3. The gaps indicate periods when the data was unavailable. The vertical right edge of Fig. 3 shows uptime per house, calculated by summing the time between gaps that are greater than one hour, and normalising this by the total monitoring duration for each house. The average uptime across all houses was 88%, with House 2 having the lowest at 76% and House 18 the highest at 94%.

The raw dataset is available on University of Strathclyde’s PURE data repository at Raw: http://dx.doi.org/10.15129/31da3ece-f902-4e95-a093-e0a9536983c4 (Data Citation 1). The cleaned dataset, where IAMs which had appliances swapped between them, have been correctly stitched together to create a continuous data stream, is also available at Cleaned: http://dx.doi.org/10.15129/9ab14b0e-19ac-4279-938f-27f643078cec (Data Citation 2). for those wishing to analyse cleaned, labelled electrical measurements immediately.

## Technical Validation

Over the course of the study there were 119,495,879 timestamped readings taken from all houses combined, with each timestamp referring to 9 appliances and an aggregate per house—leading to 1,194,958,790 readings in total. Of these, 6.4% were Not a Number (NaN) values, which represent an unchanging reading or the IAM failing to respond to requests. NaN values are still available in the Raw Data version of the dataset. In the Cleaned Data version, a notes column has been added per house to indicate when the sum of recorded IAM readings is larger than the aggregate for the corresponding sample. These are described in the ReadMe file supplied with the dataset.

All the IAM streams have been visually checked to assess the validity of the signatures which are recorded. In all cases the ReadMe file associated with the dataset accurately reflects the known appliances which were plugged in. In some cases additional signatures may appear as households have removed an appliance for a very short period of time, e.g., replacing a toaster for an infrequently used kitchen appliance.

The quality of some appliance readings is affected by the location or interference from other devices. This is more notable on appliances further from the energy aggregator as well as IAM plugs located behind devices such as washing machines and other white goods. This was detected via visual inspection against the same appliance during a similar regular usage. For example, washing machines during a spin cycle are characterised by frequent changes in power; in some cases, the power will remain static (originally NaN values which have been forward filled) for a long period of time due to a connection loss that caused a lack of updates.

Note, however, that infrequently used devices will have many NaN values only due to not being used for large periods of time, e.g., electric heaters which are typically not used during summer but left plugged in.

All IAMs exhibited erroneous spikes, some more frequently than others. There was however no correlation between appliance monitored and the number of errors which occurred. It should be noted that these errors represent less than 0.004% of total IAM readings and that across all houses there was an average of only 215 errors per IAM (over the entire 2-year monitoring period).

**Figure 4:**
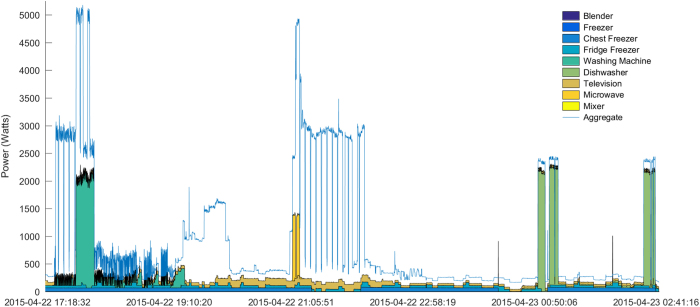
Power demand for House 10 during the evening of April 22nd 2015. The gap between the stacked IAM plot and the aggregate represents the power consumed by other appliances not monitored by IAMs.

Previously we mentioned that IAMs did not report values synchronously, as manifested by a lead with respect to the aggregate. Indeed, most IAMs should lead the aggregate by 2 or 3 readings at most. As shown in Fig. 4, typically, this issue will not affect the analysis as appliance switching on and off events can clearly be observed in the aggregate readings with a delay of up to 1–2 readings.

Meter readings were taken from several houses during installation and at subsequent visits by the REFIT project team. A comparison between the reading taken from the utility meter and the measured power by our platform is shown in Table 4. In some cases the monitored values may be higher than expected due to spikes which occurred in the aggregate. Also, as readings were only taken once every 8 s it is possible that the estimated consumption which is based on a reading multiplied by time difference to the next reading was under or over estimated. We found that the houses without solar interference had generally less than 12% difference to the utility meter estimated consumption.

Table 5 shows the % of total household consumption captured by submetering. It can be seen that up to 55% of consumed energy can be attributed to appliances directly monitored via appliance plugs. We note that the fact that some large consumers, such as the electric oven, were not monitored, resulted in a relatively low % of energy consumption captured by plugs in some houses.

In many studies such as non-intrusive appliance load monitoring (NILM) or appliance modelling, it is important to capture a large number of appliance uses. We have analysed the entire IAM dataset to record the number of uses for different appliance types. To estimate the number of uses per appliance, edge detection was used to help build up a pattern of usage.

In Table 6 we show the number of use events captured for 15 types of appliances. Note that in some cases appliances may have been monitored but used rarely. In the table, Number of Uses represents a start and end event recorded for an appliance; in the case of fridges this is a cooling cycle, e.g., from the motor starting till motor winds down to a stop. For some appliances, this number may not represent the total number of uses recorded by the REFIT study as the edge detection used was not accurate enough to classify uses in appliances where multiple devices were monitored on the same IAM, which was sometimes the case with ICT equipment (a computer, printer and monitor will be connected to the same IAM using a power strip) and Television site (Television, DVD Player, TV set-up-box were monitored together). Number of Appliances shows the total number of appliances across all REFIT households. The Pond Pump and Vivarium stayed on almost constantly, with a fixed load, throughout the study and are therefore classified as continuous use.

## Usage Notes

The data is provided in CSV format and therefore is usable in most popular software packages such as MS Excel, Matlab & SPSS. The ReadMe file explains issues with the households which have unusual wiring situations, have IAMs that were moved, or need additional processing to be used correctly.

Additionally, a NILMTK dataset converter^31^ has been created for the REFIT dataset. This was not created by the project and we cannot guarantee its functionality. It is available as part of the NILMTK program located at https://github.com/nilmtk/nilmtk.

## Additional information

**How to cite this article**: Murray, D. *et al.* An electrical load measurements dataset of United Kingdom households from a two-year longitudinal study. *Sci. Data* 4:160122 doi: 10.1038/sdata.2016.122 (2017).

**Publisher’s note**: Springer Nature remains neutral with regard to jurisdictional claims in published maps and institutional affiliations.

## Supplementary Material



## Figures and Tables

**Table 1:** Household Power/Appliance Open Access Datasets.

**Dataset**	**Location**	**Duration, Year**	**No. Houses**	**Energy Sensors**	**Data Recorded**	**Readings Freq.**
ACS-F1^32^	CHE	2*1 h sessions, 2013	N/A	100 App. (10 types)	V, I, f, P, Q, Φ	10 secs
ACS-F2^20^	CHE	2*1 h sessions, 2013	N/A	225 App. (15 types)	V, I, f, P, Q, Φ	10 secs
AMPds^16^	CAN	1 year, 2012	1	21 App.	V, I, f, pf, P, Q, S, E	1 min
AMPds2^17^	CAN	2 years, 2012	1	21 App.	V, I, f, pf, P, Q, S, E	1 min
BLUED^14^	USA	8 days, 2011	1	Agg.	V, I	12 kHz
DRED^33^	NED	6 months, 2015	1	Agg., 12 App.	P	1 Hz
ECO^22^	CHE	8 months, 2012	6	Agg., 6–10 App.	V, I, P, Q, Φ	1 Hz
GREEND^34^	AUT, ITA	1 year, 2013	9	9 App.	P	1 Hz
HES^2^	GBR	1 month (255 houses),	251	Agg., 1–10 Sub.,	P	10 min
		1 year (26 houses), 2010		13–51 App.		
iAWE^35^	IND	73 days, 2013	1	Agg., 10 App.	Agg. V, I, f, P, Q, S, E, Φ	1 Hz
					App. V, I, f, P, S, E, Φ	
IHEPCDS^18^	FRA	4 years, 2006	1	Agg., 3 Sub.	Agg. P, Q	1 min
					Sub. E	
REDD^13^	USA	3–19 days, 2011	6	Agg., 9–24 App.	Agg. V, P	Agg. 15 kHz
					App. P	App. 3 secs
REFIT	GBR	2 years, 2013	20	Agg., 9 App.	P	8 secs
Smart*^23^	USA	3 months, 2012	3	House A. Agg., 26 Sub.,	Agg. V, f, P, S	Agg. 1 Hz
				55 App.	Sub. V, f, P, S	Sub. 1 Hz
				House B, C. Agg., 21 Sub.	App. P	App. 2.5 secs
Tracebase^21^	DEU	1,883 days, 2012 onwards	15	158 App. (43 types)	P	1 Hz
UK-DALE^19^	GBR	655 days, 2012	5	Agg., 5–54 App.	Agg. V, I	Agg. 16 kHz
					App. P	App. 6 secs
Agg.=Aggregate, App.=Appliance, Sub.=Power circuit, e.g., the fuse which all appliances in a single room are connected to.						
Types is in relation to appliance groups in situations where only appliances were monitored.						
Active Power (P), Reactive Power (Q), Apparent Power (S), Energy (E), Frequency (f), Power Factor (pf), Phase Angle (Φ), Voltage (V) and Current (I). ACS-F1 and ACS-F2 datasets contain appliance signatures obtained in a laboratory setting instead of real homes.						

**Table 2:** Additional information about the houses involved in the study.

**House**	**Occupancy**	**Dwelling Age**	**# of Appliances**	**Dwelling Type**	**Size**
1	2	1975–1980	35	Detached	4 bed
2	4	—	15	Semi-detached	3 bed
3	2	1988	27	Detached	3 bed
4	2	1850–1899	33	Detached	4 bed
5	4	1878	44	Mid-terrace	4 bed
6	2	2005	49	Detached	4 bed
7	4	1965–1974	25	Detached	3 bed
8	2	1966	35	Detached	2 bed
9	2	1919–1944	24	Detached	3 bed
10	4	1919–1944	31	Detached	3 bed
11	1	1945–1964	25	Detached	3 bed
12	3	1991–1995	26	Detached	3 bed
13	4	post 2002	28	Detached	4 bed
15	1	1965–1974	19	Semi-detached	3 bed
16	6	1981–1990	48	Detached	5 bed
17	3	mid 60s	22	Detached	3 bed
18	2	1965–1974	34	Detached	3 bed
19	4	1945–1964	26	Semi-detached	3 bed
20	2	1965–1974	39	Detached	3 bed
21	4	1981–1990	23	Detached	3 bed
Occupancy column shows the number of people living in the house during the monitoring period. Number of Appliances shows the total number of electrical appliances in the house based on the conducted house survey. Size is given as number of bedrooms as this is a more common representation of dwelling size in the UK.					

**Table 3:** Monitored appliances in each house organised as shown in HES Appendix II ref. 15.

	**House Number**																				
**Appliance**	**1**	**2**	**3**	**4**	**5**	**6**	**7**	**8**	**9**	**10**	**11**	**12**	**13**	**15**	**16**	**17**	**18**	**19**	**20**	**21**	**Total**
Television	X	X	X	X	X	X	X	X	X	X		2X	2X	X	X	X	X	X	X	X	21
Hi-Fi		X							X		X							X			4
Fridge-Freezer		X	X	X	X				X	X	X	X		X	2X	X	X			X	14
Fridge	X			X			X	X			X						X	X	X		7
Freezer	2X		X	X		X	2X	X		2X						X	X	X	X		13
Microwave		X	X	X	X	X		X	X	X	X	X	X	X		X	X	X	X		16
Cooker Hood		X																			1
Kettle		X	X	X	X	X	X	X	X		X	X	X	X		X		X	X	X	16
Toaster		X	X		X	X	X	X				X		X							8
Misc Kitchen										2X								X		X	4
Washing Machine	X	X	X	2X	X	X	X	X	X	X	X		X	X	X	X	X	X	X	X	20
Washer Dryer									X								X				2
Tumble Dryer	X		X		X		X	X					X	X		X			X	X	10
Dishwasher	X	X	X		X	X	X		X	X	X		X	X	X		X		X	X	15
Computer	X			X	X	2X		X			X		X	X	X	X	X		X		13
Router											X										1
Elec Heater	X								X						2X						4
Lamp																		X			1
Misc															X	X				2X	4
The total number of appliances of the same type monitored are shown in the final column. Small/unique appliances are grouped into ‘Misc’ or ‘Misc Kitchen’ as they may only appear in one house.																					
Misc. Appliances include: House 21—Pond Pump & Vivarium, House 16—Dehumidifier, House 17—Bedroom Plug.																					
Misc. Kitchen: House 10—Mixer & Blender, House 19—Bread Maker, House 21—Mixer.																					

**Table 4:** Recorded Meter Consumption.

**House**	**Dates**	**Metered [kWh]**	**Monitored [kWh]**
8	29/09/2014–15/10/2014	226	240
8	15/10/2014–27/01/2015	1,785	1,810
8	27/01/2015–05/03/2015	657	695
10	15/10/2014–24/03/2015	2,799	2,676
13	04/10/2014–26/11/2014	640	583
17	13/11/2014–02/12/2014	178	192
18	15/10/2014–18/11/2014	333	323
18	18/11/2014–16/12/2014	316	345
19	02/12/2014–11/12/2014	79	73
Metered represents the difference in readings between the two dates which was recorded by the utility installed meter for the house. Monitored is the value calculated using the recorded data from the REFIT study.			

**Table 5:** The amount of power captured by the IAM plugs compared to the total consumption over the monitoring period.

	**House Number**																			
	**1**	**2**	**3**	**4**	**5**	**6**	**7**	**8**	**9**	**10**	**11**	**12**	**13**	**15**	**16**	**17**	**18**	**19**	**20**	**21**
% Captured by sub-metering	35	33	N/A	48	48	40	46	22	38	43	N/A	36	48	36	37	45	55	35	46	N/A
N/A value in shown for houses with solar panels.																				

**Table 6:** The amount of data collected per appliance type across all the REFIT households.

**Appliance Type**	**# of Uses**	**Consumption (kWh)**	**# of Appliances**
Fridge Freezers	121,752	20,020	13
Fridge	53,163	1,310	7
Freezers	133,967	5,486	13
Washing Machines	6,865	3,994	21
Dishwashers	4,250	6,827	14
Tumble Dryers	2,372	4,210	10
Kettle	40,092	3,298	16
Microwave	12,946	1,208	16
Toaster	5,364	257	9
ICT Equipment	4,176	3,104	13
Television Site	11,274	5,995	21
Electric Heater	503	1,023	4
Bread Maker	206	56	1
Pond Pump	Continuous	282	1
Vivarium	Continuous	208	1
